# Clinical utility of polyethylene glycol conjugated granulocyte colony-stimulating factor (PEG-G-CSF) for preventing severe neutropenia in metastatic colorectal cancer patients treated with FOLFOXIRI plus bevacizumab: a single-center retrospective study

**DOI:** 10.1186/s12885-020-06864-8

**Published:** 2020-04-28

**Authors:** Yusuke Kitagawa, Hiroki Osumi, Eiji Shinozaki, Yumiko Ota, Izuma Nakayama, Takeshi Suzuki, Takeru Wakatsuki, Mariko Ogura, Akira Ooki, Daisuke Takahari, Mitsukuni Suenaga, Keisho Chin, Kensei Yamaguchi

**Affiliations:** Department of Gastroenterology, The Cancer Institute Hospital, Japanese Foundation for Cancer Research, 3-8-31 Ariake, Koto-ku, Tokyo, 135-8550 Japan

**Keywords:** FOLFOXIRI plus bevacizumab, Neutropenia, Polyethylene glycol conjugated granulocyte colony-stimulating factor

## Abstract

**Background:**

This study aimed to evaluate the efficacy and the safety of polyethylene glycol conjugated granulocyte colony-stimulating factor (PEG-G-CSF) for preventing neutropenia in metastatic colorectal cancer (mCRC) patients that received fluorouracil, leucovorin, oxaliplatin, and irinotecan (FOLFOXIRI) plus bevacizumab (Bev) in clinical practice.

**Methods:**

We retrospectively analyzed mCRC patients who received FOLFOXIRI plus Bev between December 2015 and December 2017. We evaluated the efficacy of PEG-G-CSF as preventing or treating grade 3 or 4 neutropenia, the overall response rate (ORR) according to the Response Evaluation Criteria in Solid Tumors version 1.1, progression-free survival (PFS), overall survival (OS), and adverse events of FOLFOXIRI plus Bev based on the Common Terminology Criteria for Adverse Events version 4.0.

**Results:**

A total of 26 patients (median age 53.5 years) were included. The ORR rate was 65.3%, the median PFS was 9.6 months (7.2–16.9), and the median OS was 24.2 months (13.6–NA). Grade 3 or 4 neutropenia occurred in 53.8% of the patients, and febrile neutropenia occurred in 7.7%. PEG-G-CSF was given to 77.0% of the patients, including prophylactically (*n* = 9) and after the development of grade 3 or 4 neutropenia (*n* = 11). No patients experienced grade 3 or 4 neutropenia after the administration of PEG-G-CSF. In seven of the nine patients who received PEG-G-CSF prophylactically (77.8%), no dose adjustment was required.

**Conclusions:**

PEG-G-CSF is useful in preventing severe neutropenia in mCRC patients treated with FOLFOXIRI plus Bev.

## Background

Recently, combination chemotherapy of cytotoxic agents such as irinotecan, oxaliplatin, and fluorouracil, and molecular targeted-drugs, including anti-vascular endothelial growth factor antibody and anti-epidermal growth factor antibody have extended the overall survival (OS) of patients with metastatic colorectal cancer (mCRC) [1]. The efficacy of fluorouracil, leucovorin, oxaliplatin, and irinotecan (FOLFOXIRI) for mCRC patients in terms of overall response rate (ORR), progression-free survival (PFS), and OS was confirmed by several studies [2]. The benefit of adding bevacizumab (Bev) to the FOLFOXIRI regimen has also been demonstrated and the use of FOLFOXIRI plus Bev as an upfront treatment for mCRC patients is currently widely used [3, 4]. In the Pan-Asian adopted European Society for Medical Oncology (ESMO) consensuses guidelines, FOLFOXIRI plus Bev is recommended as first-line cytoreduction chemotherapy in “fit” mCRC patients with right-sided primary tumor location or for those with the *BRAF* V600E mutation [5]. FOLFOXIRI plus Bev is also one of the alternative treatment options of first-line chemotherapy of mCRC listed in several treatment guidelines, including the Japanese Society for Cancer of the Colon and Rectum Guidelines 2019 [6]. Furthermore, the MEBGEN RASKET™-B kit was recently approved in Japan for detecting mCRC patients with the *BRAF* V600E mutation [7]. Therefore, it is expected that the number of patients treated with FOLFOXIRI plus Bev will increase.

With regard to adverse events of FOLFOXIRI plus Bev, grade 3 or higher neutropenia or febrile neutropenia (FN) frequently occur. Several studies have shown that approximately 50% of patients experience grade 3 or higher neutropenia [3, 8–11]. In a Japanese phase 2 trial of FOLFOXIRI plus Bev for mCRC, Grade 3 or higher neutropenia and FN occurred in 72.5 and 21.7%, respectively [12]. The American Society of Clinical oncology practice guidelines recommend the prophylactic use of granulocyte colony stimulating factor (G-CSF) when the risk of FN in approximately 20% or higher [13]. Thus, we consider prophylactic G-CSF to be suitable for Japanese patients treated with FOLFOXIRI plus Bev. However, a dose adjustment of the chemotherapy is often required, and the management of neutropenia is often inadequate, even if G-CSF is administered. Polyethylene glycol-conjugated G-CSF (PEG-G-CSF), which is characterized as having an increased circulating half-life, has the potential to shorten the duration and severity of neutropenia. However, while the addition of PEG-G-CSF with FOLFOXIRI plus Bev may be useful in preventing severe neutropenia or FN, there are currently few reports evaluating the efficacy of the PEG-G-CSF for neutropenia in mCRC patients administered FOLFOXIRI plus Bev and in the safety of PEG-G-CSF administered every 2 weeks. The current study aimed to evaluate the efficacy and safety of the PEG-G-CSF for preventing neutropenia in mCRC patients treated with FOLFOXIRI plus Bev.

## Methods

### Patients

Patients diagnosed with mCRC and that received FOLFOXIRI plus Bev between December 2015 and December 2017 at the Cancer Institute Hospital, Tokyo, Japan were included in the study based on the following eligibility criteria: 1) histologically confirmed colorectal adenocarcinoma; 2) unresectable or recurrent disease; 3) no previous chemotherapy except for adjuvant chemotherapy completed more than 6 months prior to the starting date of FOLFOXIRI plus Bev treatment. The protocol summary was described on the hospital website, and the subjects were provided with the opportunity to opt-out. Therefore, no new consent for this study was required from the patients.

### Data collection

All data were collected by reviewing medical records and imaging results. We confirmed the patient age, sex, and Eastern Cooperative Oncology Group Performance Status (ECOG-PS). Data regarding the primary tumor site, the histological type of primary site tumor, whether primary resection was performed, the metastatic sites, and the number of metastatic sites were also considered. Any previous adjuvant chemotherapy, the tumor maker level before chemotherapy, *RAS* and *UGT1A1* status, the number of chemotherapy cycles, tumor response (objective response and early tumor shrinkage (ETS)), toxicity, conversion surgery rate, the date of disease progression, and the date of the last follow-up were also evaluated.

### Treatment and evaluation

Bev was administered as a 5 mg/kg intravenous dose. FOLFOXIRI treatment consisted of a 165 mg/m^2^ intravenous infusion of irinotecan for 60 min, followed by an 85 mg/m^2^ intravenous infusion of oxaliplatin given concurrently with 200 mg/m^2^ leucovorin for 120 min followed by a 3200 mg/m^2^ continuous infusion of fluorouracil for 48 h. The primary endpoint is the incidence of grade 3 or 4 neutropenia after administrating PEG-G-CSF. PEG-G-CSF (3.6 mg) starting at day four was administered every 2 weeks until progression. Whether PEG-G-CSF was used as a primary preventative treatment for neutropenia or as a secondary treatment after a patient experienced grade 4 neutropenia or FN was decided by the treating physician. In addition, the overall tumor response was assessed according to the Response Evaluation Criteria in Solid Tumors (RECIST) version 1.1 and toxicity was graded according to the Common Terminology Criteria for Adverse Events (CTCAE) version 4.0. PFS was measured as the day of initiation of FOLFOXIRI plus Bev therapy to the day on which disease progression was confirmed or to the final day of follow-up without disease progression. OS was measured as the day of initiation of FOLFOXIRI plus Bev therapy until the final day of follow-up. ETS was defined as the relative change in the sum of the longest diameters at week eight (± 4 weeks) compared to that of the baseline (cutoff: 20%).

### Statistical analysis

PFS and OS rates were estimated using the Kaplan-Meier method. All statistical analyses were performed using EZR software (Saitama Medical Center, Jichi Medical University, Saitama, Japan), which is a graphical user interface for R (The R Foundation for Statistical Computing, Vienna, Austria).

## Results

### Patient characteristics

**Fig. 1:**
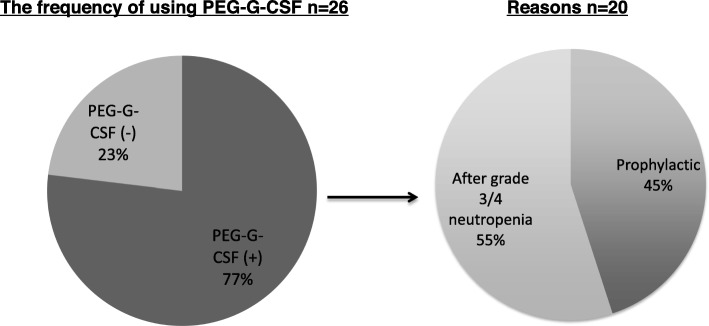
Frequency and reasons for the use of polyethylene glycol-conjugated granulocyte colony-stimulating factor (PEG-G-CSF)

**Abbreviations:** ECOG PS, eastern cooperative oncology group performance status; CEA, carcinoembryonic antigen; CA19–9, carbohydrate antigen 19–9; FN: febrile neutropenia; PEG-G-CSF: polyethylene glycol-conjugated granulocyte colony stimulating factor

The demographics and clinical characteristics of the 26 patients before the initiation of FOLFOXIRI plus Bev therapy are summarized in Table 1. Out of the 26 patients, 20 (77.0%) received PEG-G-CSF. Eleven patients received it secondarily to treat neutropenia. Among these 11 patients, 2 had previously been treated prophylactically with conventional G-CSF. Nine patients were administrated PEG-G-CSF prophylactically (Fig. 1). The median follow-up period was 24.2 months (range, 13.6-NA). The median age of the patients was 53.5 years (range, 27–74 years). Thirteen patients (50.0%) were male and 18 patients (69.2%) had an ECOG-PS of 0. The primary location of colorectal cancer was on the right side for eight (30.8%) of the patients. In addition, in the prophylactic PEG-G-CSF group, the right-sided primary tumor location was more frequent than those in the non-prophylactic PEG-G-CSF group (55.6% vs. 9.0% *P* < 0.05). The histology type was either poorly differentiated or mucinous adenocarcinoma in 5 patients (19.3%) and the primary lesion was resected in eight patients (30.8%). Metastatic lesions of the liver, lung, lymph nodes and peritoneum were detected in 23 (88.4%), 7 (26.9%), 16 (61.5%), and 5 (19.3%) of the patients, respectively. Twenty-one patients (80.7%) had two or more metastatic sites. The median carcinoembryonic antigen (CEA) and carbohydrate antigen (CA19–9) levels before chemotherapy were 88.0 ng/ml (range, 1.5–9205) and 75.4 IU/ml (range, < 2–50,000), respectively. Twenty-three patients (88.5%) had *RAS* mutation and *UGT1A1* polymorphism was observed in eight patients (30.7%).

**Table 1:** Patient demographics and clinical characteristics

Characteristics	Total (***n*** = 26) No. of patients (%)	With Prophylactic PEG-G-CSF (***n*** = 9)	Without Prophylactic PEG-G-CSF (***n*** = 11)	No use of PEG-G-CSF (***n*** = 6)
**Age at enrollment, years**				
Median	53.5	51	58	40.5
Range	27–74	34–74	27–67	35–57
**Gender**				
Male	13 (50.0)	6 (66.7)	3 (27.2)	4 (66.7)
Female	13 (50.0)	3 (33.3)	8 (72.3)	2 (33.3)
**ECOG-PS**				
/0	18 (69.2)	8 (88.9)	7 (63.6)	3 (50.0)
/1	8 (30.8)	1 (11.1)	4 (36.4)	3 (50.0)
**Primary location**				
Right	8 (30.8)	5 (55.6)	1 (9.0)	2 (33.3)
Left	18 (69.2)	4 (44.4)	10 (91.0)	4 (66.7)
**Histology**				
Diffuse	5 (19.3)	1 (11.1)	3 (27.2)	1 (16.7)
Intestinal	21 (80.7)	8 (88.9)	8 (72.3)	5 (83.3)
**Primary resection before chemotherapy**				
Yes	8 (30.8)	3 (33.3)	4 (36.4)	1 (16.7)
No	18 (69.2)	6 (66.7)	7 (63.6)	5 (83.3)
**Diagnosis of metastasis**				
Metachronous	5 (19.3)	3 (33.3)	2 (18.2)	0 (0)
Synchronous	21 (80.7)	6 (66.7)	9 (81.8)	6 (100)
**Metastatic site**				
Liver	23 (88.4)	8 (88.9)	9 (81.8)	6 (100)
Lung	7 (26.9)	3 (33.3)	4 (36.4)	0 (0)
Lymph node	16 (61.5)	2 (22.2)	8 (72.3)	6 (100)
Peritoneum	5 (19.3)	0 (0)	4 (36.4)	1 (16.7)
Other	3 (11.5)	2 (22.2)	1 (9.0)	0 (0)
**Number of metastatic sites**				
1	4 (15.4)	2 (22.2)	2 (18.2)	0 (0)
≥ 2	22 (84.6)	7 (77.8)	9 (81.8)	6 (100)
**Previous adjuvant chemotherapy**				
Yes	2 (7.7)	1 (11.1)	1 (9.0)	0 (0)
No	24 (92.3)	8 (88.9)	10 (91.0)	6 (100)
**RAS status**				
Wild type	3 (11.5)	0 (0)	2 (18.2)	1 (16.7)
Mutant type	23 (88.5)	9 (100)	9 (81.8)	5 (83.3)
**UGT1A1 Status**				
Wild type	7 (26.9)	3 (33.3)	3 (27.2)	1 (16.7)
*6	6 (23.0)	2 (22.2)	2 (18.2)	2 (33.3)
*28	2 (7.7)	0 (0)	2 (18.2)	0 (0)
Unknown	11 (42.4)	4 (44.5)	4 (36.4)	3 (50.0)
**CEA median, [range]**	88.0 [1.5–9205]	12.2 [4.6–5638]	155.9 [5–25,873]	155.9 [5–25,873]
**CA19–9 median, [range]**	75.4 [2–50,000]	99.7 [5.9–50,000]	40.2 [2.7–982]	40.2 [2.7–982]

### Adverse events, efficacy, and safety of PEG-GCSF in mCRC patients treated with FOLFOXIRI plus Bev

Grade3 or 4 toxicities of FOLFOXIRI plus Bev were shown in Table 2. The most common adverse event was hematological toxicity with grade 3 or 4 neutropenia, which was observed in 14 patients (53.8%). Grade 3 FN was observed in two patients (7.7%). Other hematological or non-hematological toxicities were less frequent such as diarrhea being observed in two patients (7.7%). No treatment-related deaths occurred. Of the 26 patients, 20 (77%) received PEG-G-CSF. None of the patients developed grade 3 or 4 neutropenia after receiving PEG-G-CSF. Ten of the 26 patients (38.5%) received a reduction in their dose of FOLFOXIRI plus Bev. Six of the 11 patients (54.6%) who received PEG-G-CSF secondarily to treat neutropenia were able to continue treating with FOLFOXIRI plus Bev without the need for a dose adjustment. On the other hand, in the 9 patients given PEG-G-CSF prophylactically, 2 (22.2%) required the dose adjustment due to non-hematological adverse events. There were no severe adverse events associated with PEG-G-CSF treatment.

**Table 2:** Toxicities according to CTCAE, version 4.0 (grade 3 or higher)

Characteristics	Total (***n*** = 26) No. of patients (%)
**Hematotoxicity**	
Neutropenia	14 (53.8)
Febrile neutropenia	2 (7.7)
Anemia	1 (3.8)
**Nonhematotoxicity**	
Infection	2 (7.7)
Nausea	1 (3.8)
Fatigue	1 (3.8)
Diarrhea	2 (7.7)
Spinal infarction	1 (3.8)
Renal dysfunction	1 (3.8)
Hypertension	1 (3.8)
Perforation	2 (7.7)

### Treatment outcomes

**Fig. 2:**
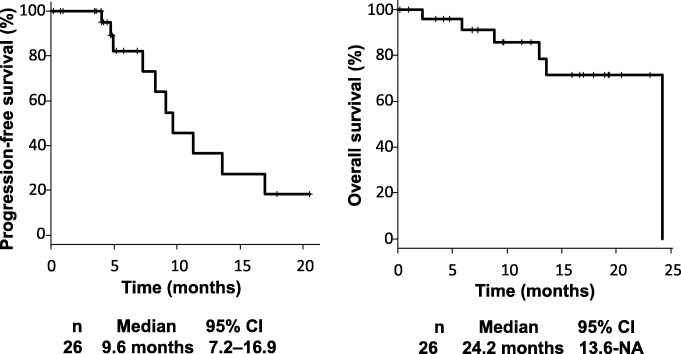
Progression-free survival and overall survival rates of the study cohort

Treatment outcomes was shown in Table 3, respectively. The median number of treatment cycles per patient was 6.5 (range, 1.0–14.0). The ORR was 65.3% (95% confidence interval [CI], 44.0–83.0) and the disease control rate was 84.5% (95% CI, 65.0–96.0). PFS and OS were 9.6 months (95% CI, 7.2–16.9) and 24.2 months (95% CI, 13.6–NA), respectively (Fig. 2). Thirteen patients (50.0%) were identified as demonstrating early tumor shrinkage, and seven patients (26.9%) received conversion surgery. As for the outcome of the patients using PEG-G-CSF, the PFSs were 4.9 and 16.9 months for the prophylactic and secondary groups, respectively (*p* < 0.05).

**Table 3:** Chemotherapeutic Efficacy

Characteristics	Total (***n*** = 26) No. of patients (%)	With Prophylactic PEG-G-CSF (***n*** = 9)	Without Prophylactic PEG-G-CSF (***n*** = 11)	No use of PEG-G-CSF (***n*** = 6)
**Number of cycles**				
Median	6.5	8	6	7
Range	1.0–14.0	3.0–10.0	5.0–14.0	1.0–11.0
**Dose reduction**				
Yes	10 (38.5)	2 (22.2)	5 (45.4)	3 (50.0)
No	16 (61.5)	7 (77.8)	6 (54.6)	3 (50.0)
**ORR**				
Partial response	17 (65.3)	3 (33.3)	10 (90.9)	4 (66.7)
Stable disease	5 (19.2)	2 (22.3)	1 (9.1)	2 (33.3)
Progressive disease	1 (3.8)	1 (11.1)	0 (0)	0 (0)
Not evaluate	3 (11.5)	3 (33.3)	0 (0)	0 (0)
**Conversion surgery**				
Yes	7 (26.9)	2 (22.2)	5 (45.4)	2 (33.3)
No	19 (73.1)	7 (77.8)	6 (54.6)	4 (66.7)
**Early Tumor Response**				
Yes	13 (50.0)	1 (11.1)	9 (81.8)	3 (50.0)
No	10 (38.4)	5 (55.6)	2 (18.2)	3 (50.0)
Not evaluated	3 (11.6)	3 (33.3)	0 (0)	0 (0)

## Discussion

In the current study, we evaluated the efficacy and safety of the PEG-G-CSF for preventing neutropenia in mCRC patients treated with FOLFOXIRI plus Bev. PEG-G-CSF prevented the development of severe neutropenia without any increases of adverse events. FN is one of the life-threatening adverse events of chemotherapy. In the 1990s, G-CSF was widely used in the clinic as a leading supportive therapy for FN. There is substantial data regarding the effectiveness of G-CSF for cancer chemotherapy [14, 15]. Compared to conventional G-CSF, the number of visits to a hospital by patients and the workload of the medical staff both decreased when we used PEG-G-CSF. This demonstrates a great benefit for the outpatient clinic.

There are several reports regarding the efficacy of PEG-G-CSF for neutropenia and FN in both mCRC and other cancers, as shown in Table 4. A Japanese double-blind placebo-controlled randomized phase 3 trial of PEG-G-CSF in 343 breast cancer patients receiving docetaxel and cyclophosphamide chemotherapy showed that the incidence of FN was significantly lower in the PEG-G-CSF group compared to that in the placebo group (1.2% vs. 68.8%, *P <* 0.001) [24]. Regarding mCRC patients, in a phase 3 double-blind trial that evaluated the efficacy of PEG-G-CSF compared to a placebo in reducing the incidence of grade 3 or 4 FN in patients with advanced CRC receiving Bev combined with first-line chemotherapy, PEG-G-CSF significantly reduced the incidence of grade 3 or 4 FN in the first four treatment cycles (PEG-G-CSF 2.4%, placebo, 5.7%, *P* = 0.014) [18]. Another randomized placebo-controlled phase 2 study examined PEG-G-CSF efficacy and safety in patients with CRC that received chemotherapy every 2 weeks. Results from this study showed that PEG-G-CSF significantly reduces the incidence of grade 3 or 4 FN (PEG-G-CSF, 2.0%; placebo, 8.0%; *P* < 0.001) [28]. Notably, this study demonstrated that PEG-G-CSF could prevent severe neutropenia in patients receiving FOLFOXIRI plus Bev on a two-week cycle without an increase of adverse events, consistent with previous reports. However, the safety of PEG-G-CSF had not been established when administered within 14 days before the start of chemotherapy. It is recommended that the administration interval of PEG-G-CSF should be 2 weeks or longer.

**Table 4:** Previous reports of efficacy of PEG-G-CSF

No.	Author	Year	Primary Tumor	Regimen	Patients number	Major outcomes
1	Yamao et al. [16]	2019	PC	mFOLFIRINOX	45	PFS (prolonged)
2	Xie et al. [17]	2018	BC	EC, TC, ET	569	Incidence and duration of grade 3/4 neutropenia
3	Pinter et al. [18]	2017	CRC	FOLFOX, FOLFIRI	845	Incidence of grade 3/4 FN in the first 4 cycles
4	Kubo et al. [19]	2016	ML	CHASE(R)	111	Duration of severe neutropenia
5	Lee et al. [20]	2016	BC	TAC	60	Duration of grade 4 neutropenia in cycle 1
6	Blackwell et al. [21]	2016	BC	TAC	308	Duration of severe neutropenia during cycle 1
7	Harbeck et al. [22]	2016	BC	TAC	316	Duration of severe neutropenia during cycle 1
8	Zhang et al. [23]	2015	BC	TAC	171	Incidence of grade 3/4 neutropenia
9	Kosaka et al. [24]	2015	BC	TC	351	Incidence of FN
10	Bozzoli et al. [25]	2015	DLBCL	RCHOP	51	Frequency of FN and unplanned hospitalizations
11	Gladkov et al. [26]	2015	BC	Doxorubicin/Docetaxel	78	Incidence of adverse events
12	Shi et al. [27]	2013	BC, NSCLC, NHL, HNC	PC, AC, CHOP	337	Rate of protection against grade 4 neutropenia
13	Hecht et al. [28]	2010	CRC	FOLFOX, FOLFIRI, FOIL	241	Incidence of grade 3/4 neutropenia.
14	Fox et al. [29]	2009	Sarcoma	VDC, IE	34	Duration of severe neutropenia
15	Sierra et al. [30]	2008	AML	Idarubicin/cytarabine	84	Assisting neutrophil recovery
16	von Minckwitz et al. [31]	2008	BC	TAC	1256	Primary prophylaxis of FN and related toxic effects
17	Bladucci et al. [32]	2007	Solid tumors or NHL	Carboplatin, Cisplatin, Doxorubicin, Doxorubicin and Paclitaxel, AC, Docetaxel, ACT, FEC, CHOP, EPOCH, Topotecan	852	Proportion of patients experiencing FN
18	Romieu et al. [33]	2007	BC	FEC	60	Incidence of neutropenic events
19	Vogel et al. [34]	2005	BC	Docetaxel	928	Percentage of patients developing FN
20	Grigg et al. [35]	2003	NHL	CHOP	50	Duration of grade 4 neutropenia
21	Vose et al. [36]	2003	ML	ESHAP	66	Incidence of grade 4 FN
22	Green et al. [37]	2003	BC	DA	157	Incidence of Grade 4 neutropenia
23	Holmes et al. [38]	2002	BC	DA	310	Absolute neutrophil count
24	Holmes et al. [39]	2002	BC	DA	154	Incidence of Grade 4 neutropenia in cycle 1
25	Johnston et al. [40]	2000	NSCLC	Carboplatin and Paclitaxel	13	Serum concentrations

In addition, *UGT1A1* polymorphism was detected in this study in eight (30.7%) of the patients (*6 in six patients, *28 in two patients). Among these patients with *UGT1A1* polymorphism, six had been administered PEG-G-CSF, two after the development of grade 3 neutropenia, and four prophylactically. In Japan, the incidence of *UGT1A1* *6 polymorphism is higher than that in the US and European countries [41–43]. In a Japanese phase 2 trial of FOLFOXIRI plus Bev in mCRC patients, the frequency of neutropenia in patients with *UGT1A1* *6 or *28 polymorphism is higher than that in patients with wild-type *UGT1A1*^10^. However, in the current study, no patients experienced severe neutropenia after the administration of PEG-G-CSF, even those with *UGT1A1* *6 or *28 polymorphism. Furthermore, 5 of the 6 patients could continue the FOLFOXIRI plus Bev treatment without any need for a dose adjustment. These data suggest that the administration of PEG-G-CSF with a two-week cycle may be safe and PEG-G-CSF can prevent severe neutropenia in patients with *UGT1A1* *6 or *28 polymorphism.

There were several limitations of our study. Firstly, this was a retrospective study with relatively small sample size. Secondly, PFS was significantly different between the prophylactic and non-prophylactic PEG-G-CSF groups. This difference was partially because the mCRC patients in the prophylactic PEG-G-CSF group mostly had the tumor on the right side, rather than the left, and this sub-group has poorer survival than the patients with the tumor on the left. Therefore, further research is necessary to evaluate the correlation between the timing of PEG-G-CSF use (prophylactic or non-prophylactic) and survival. However, even with these limitations, the results of this study showed that neutropenia, which is the most common adverse event in patients under treatment with FOLFOXIRI plus Bev, could be prevented by using PEG-G-CSF.

**Abbreviations:** PC, pancreatic cancer; BC, breast cancer; CRC, colorectal cancer; ML, malignant lymphoma; DLBCL, diffuse large B-cell lymphoma; NSCLC, non-small cell lung carcinoma; NHL, non-Hodgkin’s lymphoma; HNC, head and neck carcinoma; AML, acute myeloid leukemia; mFOLFIRINOX, modified fluorouracil, leucovorin, oxaliplatin, and irinotecan: EC, epirubicin and cyclophosphamide; TC, Taxotere and cyclophosphamide; ET, endocrine therapy; CHASE(R), cyclophosphamide, cytarabine, dexamethasone, etoposide (and rituximab); DA,; TAC, taxotate, adriamycin and cyclophosphamide, (R) CHOP, rituximab, cyclophosphamide, doxorubicin, vincristine and prednisolone; PC, paclitaxel and carboplatin; AC, adriamycin and cyclophosphamide; VDC, vincristine, doxorubicin and cyclophosphamide; IE, fosfamide and etoposide; ACT, doxorubicin, cyclophosphamide and docetaxel; FEC, falmorubicin, endoxane and 5-fluorouracil; EPOCH, etoposide, prednisolone, vincristine, cyclophosphamide and doxorubicin; ESHAP, prednisolone, etoposide, cytarabine and cisplatin; DA, docetaxel and doxorubicin;

## Conclusion

PEG-G-CSF is useful for both primary and secondary prevention of severe neutropenia in mCRC patients treated with FOLFOXIRI plus Bev without increases in adverse events.

## Data Availability

All data generated or analyzed during this study are included in this published article.

## Abbreviations

PEG-G-CSF: Polyethylene glycol conjugated granulocyte colony-stimulating factor
mCRC: Metastatic colorectal cancer
FOLFOXIRI: Fluorouracil, leucovorin, oxaliplatin, and irinotecan
Bev: Bevacizumab
ORR: Overall response rate
PFS: Progression-free survival
OS: Overall survival
ESMO: European Society for Medical Oncology
FN: Febrile neutropenia
ECOG-PS: Eastern Cooperative Oncology Group Performance Status
ETS: Early tumor shrinkage
RECIST: Response Evaluation Criteria in Solid Tumors
CTCAE: Common Terminology Criteria for Adverse Events
CEA: Carcinoembryonic antigen
CA19–9: Carbohydrate antigen
PC: Pancreatic cancer
BC: Breast cancer
CRC: Colorectal cancer
ML: Malignant lymphoma
DLBCL: Diffuse large B-cell lymphoma
NSCLC: Non-small cell lung carcinoma
NHL: Non-Hodgkin’s lymphoma
HNC: Head and neck carcinoma
AML: Acute myeloid leukemia
mFOLFIRINOX: Modified fluorouracil, leucovorin, oxaliplatin, and irinotecan
EC: Epirubicin and cyclophosphamide
TC: Taxotere and cyclophosphamide
ET: Endocrine therapy
CHASE(R): Cyclophosphamide, cytarabine, dexamethasone, etoposide (and rituximab)
DA: Docetaxel and doxorubicin
TAC: Taxotate, adriamycin and cyclophosphamide
(R)CHOP: (Rituximab), cyclophosphamide, doxorubicin, vincristine, and prednisolone
PC: Paclitaxel and carboplatin
AC: Adriamycin and cyclophosphamide
VDC: Vincristine, doxorubicin, and cyclophosphamide
IE: Fosfamide and etoposide
ACT: Doxorubicin, cyclophosphamide, and docetaxel
FEC: Falmorubicin, endoxane and 5-fluorouracil
EPOCH: Etoposide, prednisolone, vincristine, cyclophosphamide, and doxorubicin
ESHAP: Prednisolone, etoposide, cytarabine and cisplatin
